# Short‐term exposure to urban PM_2.5_ particles induces histopathological and inflammatory changes in the rat small intestine

**DOI:** 10.14814/phy2.15249

**Published:** 2022-04-13

**Authors:** Lena Ohlsson, Christina Isaxon, Sebastian Wrighton, Wissal El Ouahidi, Lisa Fornell, Lena Uller, Saema Ansar, Ulrikke Voss

**Affiliations:** ^1^ 5193 Unit of Experimental Vascular Research Department of Clinical Sciences Lund University Lund Sweden; ^2^ 5193 Division of Ergonomics and Aerosol Technology Department of Design Sciences Lund University Lund Sweden; ^3^ 5193 Division of Infection Medicine Department of Clinical Sciences Lund University Lund Sweden; ^4^ 5193 Unit of Applied Neurovascular Research Department of Clinical Sciences Lund University Lund Sweden; ^5^ 5193 Unit of Respiratory Immunopharmacology Department of Experimental Medical Sciences Lund University Lund Sweden

**Keywords:** environmental, gastrointestinal, inflammation, physiology, toxicology, urban air pollution

## Abstract

Air pollution and exposure to fine airborne particles with aerodynamic diameter <2.5 μm (PM_2.5_) negatively impacts human health. Airways constitute a primary route of exposure but PM_2.5_‐contaminated food, drinks as well as mucociliary and hepatobiliary clearance all constitute potential entry points into the intestine. This study evaluated intestinal histopathological and inflammatory changes as well as enteric neuronal numbers after short‐ or long‐term exposure to urban PM_2.5_. Using a nebulizer, male rats were exposed to a mist with a concentration of 5.3mg PM_2.5_/m^3^ for 8 h (short term) or 1.8 mg PM_2.5_/m^3^ for 3 h/day, 5 days/week for 8 weeks (long‐term) with controls run in parallel. Samples were taken from three regions of the small intestine as well as the colon. Results showed that short‐term exposure to PM_2.5_ induces mucosal lesions and reduces IL1β levels in the small intestine but not colon. No significant changes were observed after long‐term exposure, suggesting the presence of intestinal adaptation to environmental stressors in the PM_2.5_. To our knowledge, this is the first study to systematically characterize regional effects along the intestine.

## Graphical abstract

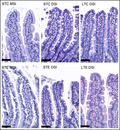

## 1 INTRODUCTION

Air pollution is the fifth ranking mortality risk factor, accountable for more than 7% of global deaths yearly, particularly in low‐ and middle‐income countries (Cohen et al., 2017). These detrimental health effects have, in particular, been associated with fine airborne particles with an aerodynamic diameter of <2.5 μm (PM_2.5_) (Cohen et al., 2017). These are a byproduct of human activity, which beyond soot from combustion, can contain metals including lead and arsenic (Nääv et al., 2020). Moreover, organic pollutants such as the polycyclic aromatic hydrocarbons (PAH) bezo(a)pyrene, a potent carcinogenic can adsorb to PM_2.5_ (Leung et al., 2014; Nääv et al., 2020).

PM_2.5_ exposure has been linked to several diseases including pulmonary, cardiovascular, and metabolic (Cohen et al., 2017; Li et al., 2019). While the World Health Organization (WHO) recommends that PM_2.5_ exposure should on an annual average not exceed 5 μg/m^3^ (Health Effects Institute, 2020), which based on a respiratory capacity of 6 L/min correlates to an airway exposure of ~1.8 μg/h. Measurements conducted in five Chinese cities found that the PM_2.5_ peak burden exceeded 300 μg/m^3^ (Leung et al., 2014), correlating to an airway exposure above 100 μg/h. Further, the latest report released by the health effects institute shows that none of the four world regions meet the WHO recommendations (Health Effects Institute, 2020). The respiratory tract constitutes the main route of exposure where PM_2.5_, due to its small size, can pervade affected lungs causing local cell damage and low‐grade chronic inflammation (Falcon‐Rodriguez et al., 2016). Continuous PM_2.5_ exposure alters the physiological immune response, rendering lung tissue susceptible to disrupted barrier integrity and infections (Feng et al., 2000, 2016). While the majority of the work done focuses on airways, PM_2.5_ contaminated food, drinks, as well as mucociliary and hepatobiliary clearance of PM_2.5_ all constitute potential intestinal exposure. Particle size largely determines whether particles are able to reach airways, for a particle to be respirable and pass the larynx it needs a diameter of 10 μm or less (Brown et al., 2013). Studies of mucociliary clearance using spheres of different sizes have shown that 70% of 6 μm spheres placed in the nasal cavity of rats are cleared to the gastrointestinal tract (GIT) by mucociliary transport within 15 min (Inoue et al., 2018). Further that 60%–80% of 1 μm and 10 μm spheres instilled into the airways were cleared by mucociliary transport within 4 h (Coote et al., 2004).

The intestinal epithelial lining consists of cells which allow for the absorption of nutrients, regulate appetite, and act as a barrier between the microbiome and the body. While the gut associated lymphoid tissue is under normal circumstances immunological tolerant toward irritants such as microbial and nutritional compounds present in the lumen, an imbalanced gut microbiome has been extensively linked with inflammation (Lo et al., 2020), potentially sparking the genesis of diseases such as inflammatory bowel disease, obesity and type 2 diabetes (Jackson et al., 2018). In mice, airway exposure to PM_2.5_ has been found to alter the microbiome (Bailey et al., 2020; Ran et al., 2021). Air pollution has also been associated with increased oxidized lipids and inflammatory changes including altered villus morphology and infiltration of neutrophils and macrophages (Feng et al., 2020; Ran et al., 2021).

In this study, we have evaluated intestinal histopathology and inflammation following short‐ and long‐term inhalation exposure with its associated secondary intestinal exposure to well‐characterized urban PM_2.5_ by exposing phenotypically normal rats to an aerosolized mist.

## 2 METHODS AND MATERIALS

### 2.1 Urban PM_2__.__5_ particles

Particle collections were performed during 26 days from April to May. The collector, a high‐volume cascade impactor (HVCI) (BGI900, Mesa Labs, USA) was placed at a height of 3.4 m from a street crossing with average daily traffic of 28,000 vehicles in the center of the southern Swedish city of Malmö. The HVCI samples air (0.9 m^3^/min) and collects PM_2.5_ on a Teflon filter. A tapered element oscillating microbalance (TEOM 1400AB) was used for time resolved PM_2.5_ mass concentration measurements and aethalometer (AE33) for assessment of black carbon (soot) and organic carbon by light absorption. PM_2.5_ were extracted from the filters using analysis grade methanol (MeOH) according to Mesa Labs’ protocol (Cassee et al., 2003). The solution was pipetted into pre‐cleaned glass vials and dried in a vacuum evaporator (SpeedVac HT‐4X Evaporator, GeneVac, UK). The dried collected particles were analyzed by gas chromatography–mass spectrometry (GC–MS) for PAH content (Kliucininkas et al., 2011), and by inductively coupled plasma mass spectrometry (ICP‐MS) for metal composition using previously described protocols (Nääv et al., 2020) at the Department of Occupational and Environmental Medicine at Lund‐University, Sweden.

### 2.2 Animals

Male Sprague–Dawley rats (*n* = 28, 240‐260 g Janvier labs, FR) approximately 2 months old and estimated to have reached young adulthood (Sengupta, 2013), were housed in pairs and used in the experiments. Animals had free access to chow (Lactamin R36, SE) and water. Air in the facilities is ventilated through HEPA filters and cages contain sterilized wood bedding. Animals had at least a week of acclimatization before the start of the experiments. All experiments were performed during the light cycle.

### 2.3 PM_2__.__5_ delivery

Dried PM_2.5_ particles were dispersed in phosphate buffered saline (PBS) and sonicated in an ultrasonication water bath (22–25°C) for 30 min before each inhalation experiment. PBS only (without added PM_2.5_) control exposure experiments were run in parallel. For short‐term exposures, a solution of 7 mg PM_2.5_ per 1L PBS was prepared, for long‐term exposures, a solution of 2.4 mg PM_2.5_ per 1L PBS was prepared. The dispersed PM_2.5_ particles as well as the control PBS solution were delivered using jet‐nebulizers (500 ml Inline Micronebulizer, Bird Co. USA) with a fixed pressure of 4 Bar (Alenmyr et al., 2005), creating a flow concentration of 240 μg/h and 80 μg/h for short‐ and long‐term experiments, respectively. The aerosolized solutions were delivered into two Plexiglas exposure chambers each (dimensions of each chamber 16 × 15.5 × 20 cm) through clear plastic tubing (L 60 cm, Ø 1 cm). The flow into each chamber was 16.5 ml/h with a steady state between mist into and out of chambers achieved after 15 min. Providing a PM_2.5_ mist concentration at steady state of 5.3 mg/m^3^ in short‐term and 1.8 mg/m^3^ in long‐term experiments, concentrations well above WHO guidelines and recorded peak levels. This concentration was needed to achieve estimated airway concentrations of 90 μg/h for short‐term exposure and 30 μg/h for long‐term exposures. Estimation based on unstressed breathing and a ventilation capacity of 0.4 m^3^ a day in rats (Office of Environmental Health Hazard Assessment California Environmental Protection Agency, 2018). However, actual alveolar exposure might be significantly reduced by nasal and tracheobronchial mucociliary capture and clearance of PM_2.5_. This effect is estimated to be approximately 70% each (Coote et al., 2004; Inoue et al., 2018) if reaching these levels in the current study it would lead to an actual alveolar exposure of 8.1 μg/h and 2.7 μg/h in short‐ and long‐term, respectively. With remaining particles cleared to the GIT at 82 μg/h and 27 μg/h for short‐ and long‐term, respectively. Each exposure chamber held two animals enabling the treatment of four animals from each experimental group to be run in parallel. Animals that were housed together also shared exposure chambers to reduce stress.

### 2.4 Exposure setup

Two exposure protocols were evaluated: A short‐term peak concentration exposure and a long‐term above‐recommendations exposure protocol. After each exposure session, the animals were returned to their home cages.

In the short‐term exposure (STE) experiments, animals were exposed once to a mist with a concentration of 5.3 mg/m^3^ PM_2.5_ for 8 h (*n* = 6), with an estimated gastrointestinal load of ~646 μg/session. Short‐term control (STC) animals (*n* = 6) received PBS and were run in parallel. The animals were anesthetized and sacrificed by aortic puncture 44 h after exposure. In the long‐term exposure (LTE) experiments animals (*n* = 8) were exposed to a mist with a concentration of 1.8 mg/m^3^ PM_2.5_ for 3 h/day, 5 days/week for 8 weeks, with an estimated gastrointestinal load of ~81 μg/session. Long‐term control (LTC) animals (*n* = 8) received PBS and were run in parallel. After the last exposure, the animals were anesthetized and sacrificed by aortic puncture.

### 2.5 Sample collection

The animals were anesthetized by intraperitoneal injection of pentobarbital sodium (0.5 ml/100 g), their abdominal cavity opened, saline moistened gauze was used to push aside internal organs and expose the aorta which was punctured. The GIT was removed from the lower esophageal sphincter to the distal colon and placed in chilled Hank's balanced salt solution (HBSS, gibco™, Thermo Fisher Scientific, SE). The proximal small intestine (PSI) was defined 3–6 cm below the pyloric sphincter, the middle small intestine (MSI) was defined as 13–16 cm below the pyloric sphincter, the distal small intestine (DSI) was defined as the last 3–4 cm before the ileocecal sphincter and the colon (Col) was defined as 2 cm below the cecum. Sections of PSI, MSI, DSI, and Col were collected from each animal and rinsed in cold HBSS. One section was moved to a 0.5‐ml plastic tube containing RNA later (AM7021, Thermo Fisher Scientific, SE) and stored at −80°C for later analysis. Another larger part was opened along the mesenteric border, oriented for longitudinal sectioning, and placed between two filter papers before being immersion fixed over‐night at 4°C in 4% paraformaldehyde in 0.1 M phosphate buffer. Embedding was preceded by three rinses in 70% ethanol followed by dehydration, clearing, and embedding. Paraffin‐embedded samples were sectioned at 4 μm on a microtome for histological and immunocytochemical analyses. Sections were deparaffinized by two washes in xylene followed by rehydration through stepwise washes in graded series of ethanol and water.

### 2.6 Histological assessment

Rehydrated tissue sections were hematoxylin and eosin stained for histopathological analysis. In brief, sections were submerged 10 min in Mayer's hematoxylin, followed by 10 min wash under running tap water. This was followed by 7 min submersion in eosin. Lastly, slides were washed 2 min under running tap water followed by dehydration by stepwise washes in graded series of ethanol and xylene before being mounted in Pertex™ (Histolabs, SE). Slides were scanned on either a ScanScope CS2 (Aperio Leica systems, LRI, SE) or a Nanozoomer 2HT (Hamamatsu, JP) in brightfield mode with 20x objective. Slides were analyzed using QuPath software (Bankhead et al., 2017). Lesion scores for tunica muscularis and mucosa were analyzed as described by De Ceulaer et al. (De Ceulaer et al., 2011). Mucosal damage in the small intestine was assessed on a grading scale from 0 to 5, where: 0 = no lesions present; 1 = a small subepithelial space at the villus tip is present; 2 = the lamina propria is exposed at the tips and epithelial cells are detached from the lamina propria; 3 = epithelial cells are separated from the underlying lamina propria extending as far as halfway down the villus; 4 = the lamina propria is stripped to the base of the villus; and 5 = the lamina propria and mucosa suffer from a total loss of structural integrity. Colonic mucosal lesions were graded on a score of 0–6 dependent on the presence of disrupted crypt structure, mucosal bleeding, and immune cell infiltration, where 0 indicates “not present”; 2, “sporadically present”; 4, “present”; 6, “abundantly present”. Tunica muscularis damage was assessed by assessing the presence of intramural bleeding, infiltration of inflammatory cells, vacuolar degeneration, and wavy fibers individually on the same 0–6 scale as above. Representative micrographs of the histological assessment scores can be found in Figure S1.

### 2.7 Immunocytochemical assessment of enteric neurons

Rehydrated sections were subjected to antigen retrieval by microwaving 2 × 8 min at 650 W in citric acid buffer (0.01 M, pH 6) followed by 10 min cooling in citric acid buffer and 20 min washing under cold tap water. Washed slides were incubated 10 min in 3% hydrogen peroxide prior to 10 min washing in PBS supplemented with 0.25% Triton X100 (PBS‐T). Slides were incubated over‐night at 4°C with primary antibody against the pan neuronal marker HuC/HuD (A‐21271, Thermo Fisher Scientific, SE, RRID AB_221448, (Cheng et al., 2016)) diluted 1:400 in PBS with 0.25% BSA. Sections were washed 3 × 10 min in PBS‐T followed by 1 h incubation with SignalStain boost‐detection HRP‐mouse (8125S, Cell Signaling, USA, RRID AB_10547893). Slides were washed 3 × 10 min in PBS‐T followed by development for 1 min with DAB reagent (sk‐4100, Vector, USA, RRID AB_2336382). Slides were washed 10 min under running tap water before being submerged 30 s in hematoxylin for counterstaining. Following counterstaining slides were washed 10 min under running tap water and dehydrated by stepwise washes in increasing grades of ethanol and xylene before being mounted with Pertex™ (Histolab, SE). Slides were scanned on a Nanozoomer 2HT (Hamamatsu, JP) in brightfield mode with 20x objective and analyzed using QuPath software (Bankhead et al., 2017).

### 2.8 Cyto‐and chemokine analyses

Protein was extracted from intestinal samples stored in RNA‐later at −80°C. During extraction, all reagents and samples were kept on ice. Thawed samples were weighed and protein extraction buffer containing RIPA (899001, Thermo Fisher Scientific, SE), 1% protease inhibitor (Halt 78430, Thermo Fisher Scientific, SE), 1% 0.5 M EDTA, and 0.04% deoxyribonuclease I (Merck, SE), were added at 10 µl per 1 mg of tissue. Samples were homogenized using Potter‐Elvehjem type tissue grinders. Homogenized samples were centrifuged at 4°C and 13000 rpm for 10 min. Supernatants were collected and stored at −20°C until further analysis. Protein content was measured in each sample using the BCA protein assay kit (Pierce 23227, Thermo Fisher Scientific, SE) according to manufacturer's protocol. Absorbance was measured at 580 nm using the FLUOstar Omega Microplate Reader (BMG Labtech, SE). Cyto‐ and chemokine concentration in the samples were measured using the proinflammatory electrochemiluminescence multi‐spot assay kit (#K15059D‐1, Mesoscale Discovery Systems, USA) which detects 9 cyto‐ and chemokines Interleukin (IL)1β, IL4, IL5, IL6, IL10, IL13, interferon γ (INFγ), c‐x‐c chemokine ligand 1 (CXCL1), and tumor necrosis factor (TNF). Analyses were carried out in accordance with the manufacturer's instructions and measured on a Meso‐Quickplex sq 120 platform. All samples and standard series were run in duplicates and concentrations were normalized to protein content.

### 2.9 Analyses

IL5 were in ≈67% of all long‐term samples below analysis range across all intestinal regions and have been excluded. Further, ≈48% of IL13 samples in PSI and MSI were below the detection rate and have therefore been excluded. Histological and immunocytochemical analyses were evaluated blinded to treatment. Histological assessments were based on 6–9 nonconsecutive tissue sections per area and animal. Morphometric measurement of tunica muscularis and mucosal thickness were done on areas with an intact morphology, including clear smooth muscle layer definition and intact crypt‐villi, alternatively crypt structure. An average of 8 measurements were taken from each section. Enteric neuronal numbers were counted from an average of 9 mm longitudinal cut intestinal sections. Two‐way ANOVA was used to assess for differences across and within intestinal area. For comparisons of short‐ and long‐term effects across intestinal areas, Turkey's multiple comparisons analysis was used. For comparisons between PM_2.5_‐exposed groups and their respective controls within intestinal area multiple comparisons using the Holm–Sidak analysis was used. All analyses were done in Prism 8.3.0 (GraphPad, USA). Some samples were lost during embedding procedures, for example, circular mounted tissues. Samples measured to be outside the detection range in the multiplex analysis were excluded, leading to reduced group sizes in some of the analyzed parameters. Individual values and mean ± SD are presented in all figures.

## 3 RESULTS

Details on the content of the PM_2.5_ can be found in supplementary information in Nääv et al. (2020).

### 3.1 General observations

Animals remained healthy throughout the experiments, while in the exposure chambers animals initially exhibited explorative behaviors but familiarized quickly. During the exposure period animals mainly slept or exhibited grooming behavior, this was likely due to the mist filling the chamber rendering animals damp.

### 3.2 Histopathological changes after PM_2__.__5_ exposure

**FIGURE 1:**
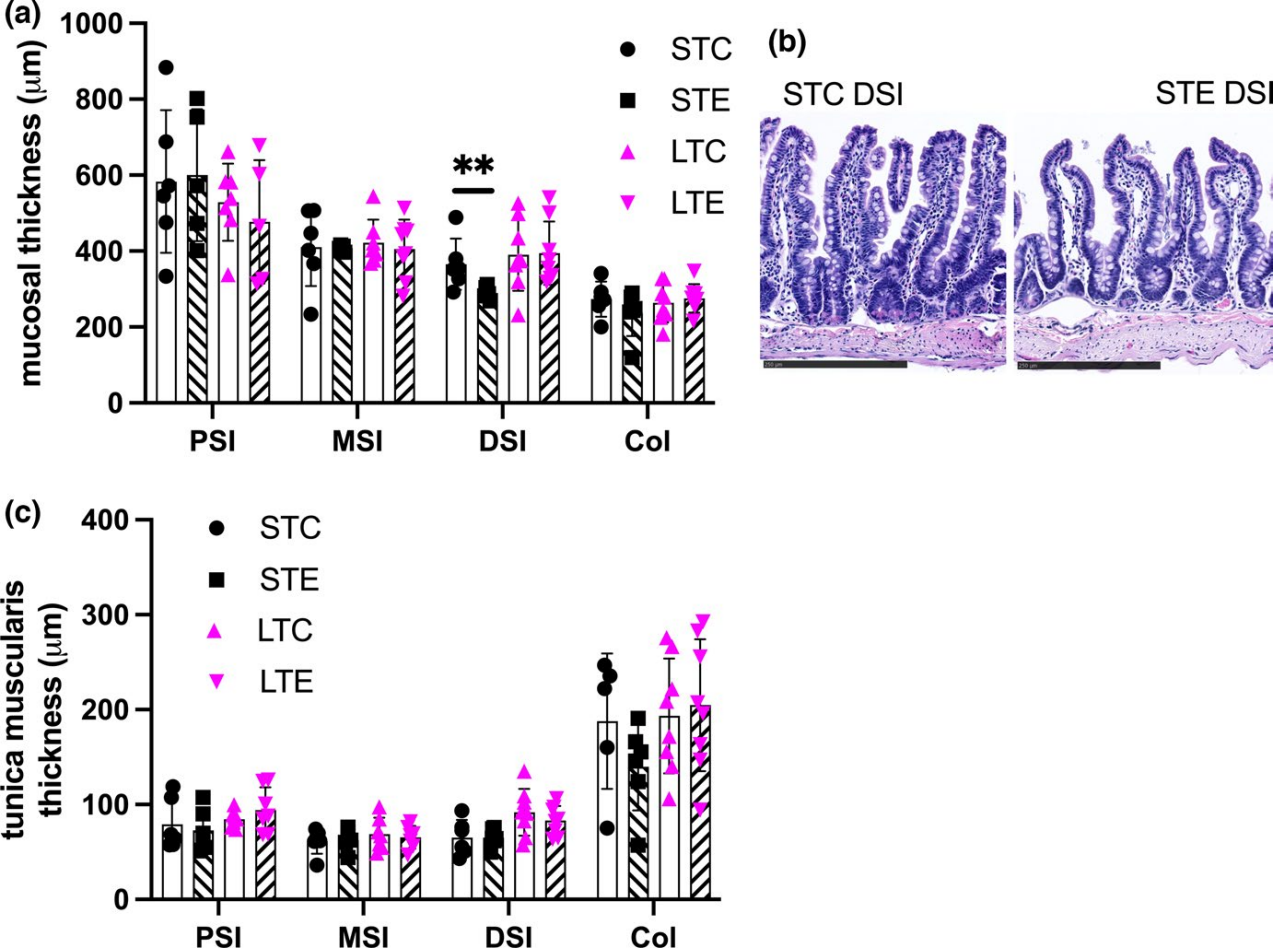
Short‐term PM_2.5_ exposure reduces crypt‐villus length in distal small intestine (DSI). (a) Mucosal thickness of intact crypt‐villus and crypts along the intestine in after short‐term (ST) and long‐term (LT) exposure to PM_2.5_ dispersed in PBS (STE, LTE; indicated with hatched bars) or PBS exposed controls (STC, LTC, indicated with white bars). (b) Representative micrographs of DSI mucosa showing reduced crypt‐villus height in short‐term‐exposed animals. (c) Tunica muscularis thickness along the intestine. Data presented as individual values and mean ± SD, ^**^ *p* < 0.01, bar in micrographs represents 250 μm. PSI, proximal small intestine; MSI, middle small intestine; DSI; Col, colon

**FIGURE 2:**
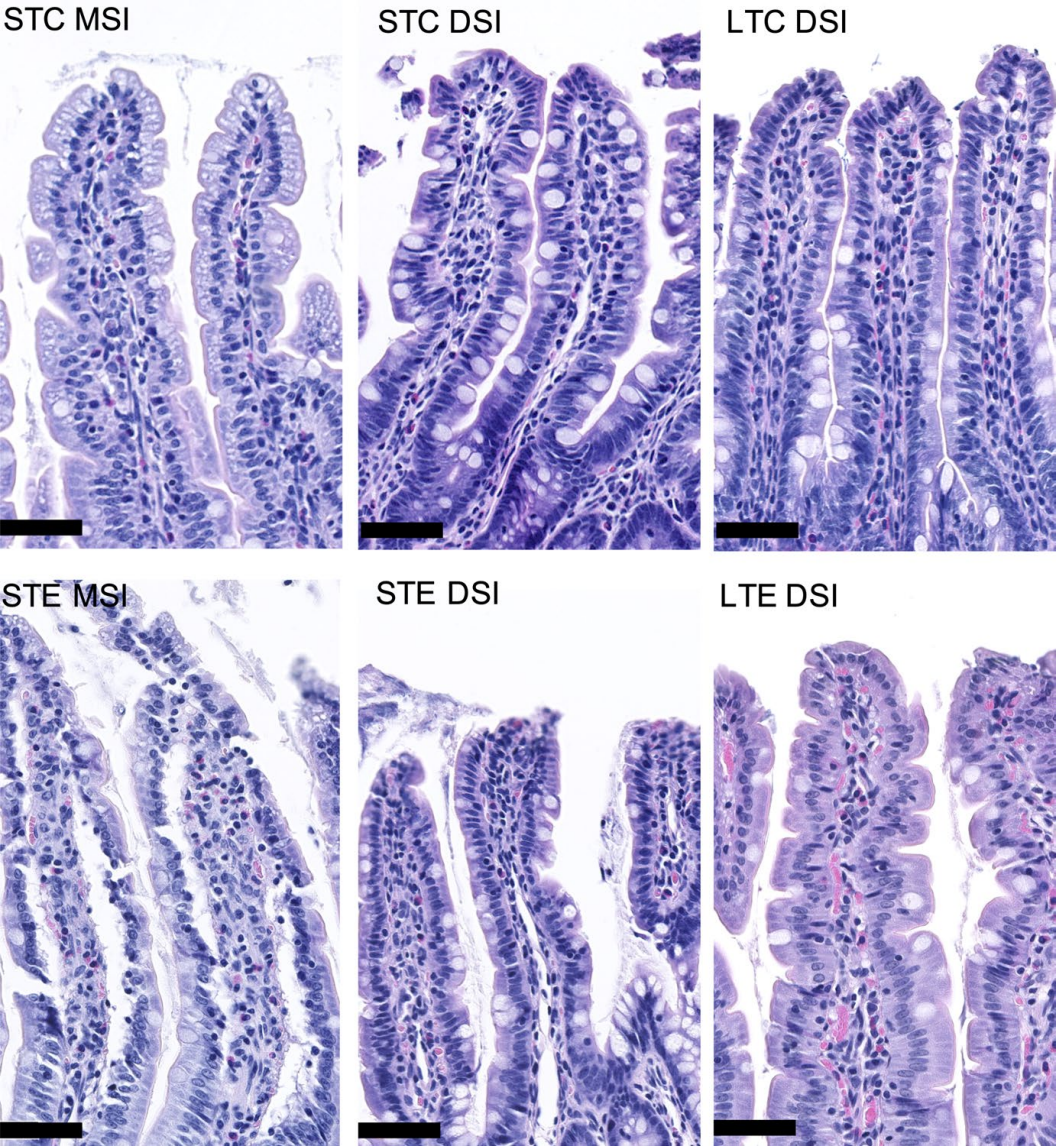
Short‐term PM_2.5_ exposure induce mucosal lesions in the middle (MSI) and distal small intestine (DSI). Top row representative micrographs of short‐term control (STC) mucosa in MSI and DSI and long‐term control (LTC) mucosa in DSI. Bottom row representative micrographs of mucosal lesions in MSI and DSI after short‐term PM_2.5_ exposure (STE), and increased mucosal blood infiltration in DSI after long‐term PM_2.5_ exposure (LTE). Bar represents 50 μm

Gross analyses of intact crypt‐villus and crypt length showed short‐term PM_2.5_ exposure to reduce crypt‐villus length compared to short‐term control in DSI but not in other intestinal regions (*p* = 0.009, Figure 1a,b). No effects of PM_2.5_ exposure compared to their respective controls were found on the thickness of tunica muscularis (Figure 1c). Analyses of selected histopathological parameters showed short‐term exposure to increase mucosal lesion score in MSI and DSI, this was absent in the long‐term‐exposed animals. With MSI showing less mucosal lesions after long‐term exposure. Long‐term exposure was found to increase the presence of mucosal blood in the DSI. No significant change in the evaluated histopathological parameters was observed in the tunica muscularis across intestinal regions and treatments. Results are presented in Tables 1 and 2 with representative control and lesion‐associated micrographs in Figure 2.

**TABLE 1:** Determination of mucosal lesions along the GIT for control and PM_2.5_‐exposed animals

Lesion type (score range)	Tissue	STC mean ± SD	STE mean ± SD	LTC mean ± SD	LTE mean ± SD
Villus integrity (0–5)	PSI	1.0 ± 0.6	1.7 ± 1	2.2 ± 0,3	2.2 ± 0.8
	MSI	1.4 ± 1	3.0 ± 0.4**	1.8 ± 1	0.4 ± 0.3*
	DSI	0.4 ± 0.3	1.9 ± 0.9*	0.9 ± 0.6	1.5 ± 0.6
Crypt integrity (0–6)	Col	2.3 ± 2	1.5 ± 2	2.2 ± 1	1.6 ± 1
Blood (0–6)	PSI	2.8 ± 2	4.0 ± 0.8	2.3 ± 0.6	2.0 ± 1
	MSI	2.5 ± 2	1.8 ± 1	2.0 ± 1	0.8 ± 0.5
	DSI	2.3 ± 2	2.0 ± 2	1.0 ± 0.8	2.8 ± 2*
	Col	1.5 ± 1	2.3 ± 2	1.1 ± 1	1.4 ± 1
Immune infiltration (0–6)	PSI	3.8 ± 1	4.8 ± 0.5	4.0 ± 0	3.0 ± 1
	MSI	3.5 ± 0.8	5.0 ± 0.8	3.5 ± 0.6	1.8 ± 1
	DSI	3.0 ± 1	4.0 ± 0.8	3.6 ± 2	3.3 ± 1
	Col	3.5 ± 1	3.0 ± 1	2.8 ± 1	2.6 ± 2
Eosinophilic infiltration (0–6)	PSI	2.5 ± 2	3.5 ± 0.6	4.7 ± 0.6	4.0 ± 0
	MSI	3.0 ± 1	3.5 ± 0.6	4.0 ± 1	3.0 ± 1
	DSI	2.0 ± 1	1.0 ± 2	2.5 ± 1	2.4 ± 1
	Col	0.5 ± 0.6	0.3 ± 0.5	1.1 ± 0.8	0.9 ± 0.7

Abbreviations: Col, colon (n_ST_ = 3–4 n_LT_ = 8); DSI, distal small intestine (n_ST_ = 4 n_LT_ =8); MSI, middle small intestine (n_ST_ = 4 n_LT_ = 4); PSI, proximal small intestine (n_ST_ = 4 n_LT_ = 3); SD, standard deviation, ^*^ *p* < 0.05, ^**^ *p* < 0.01.

**TABLE 2:** Determination of tunica muscularis lesions and their histopathological scores along the GIT for control and PM_2.5_‐exposed animals

Lesion and score	Tissue	STC mean ± SD	STE mean ± SD	LTC mean ± SD	LTE mean ± SD
Intramural blood (0–6)	PSI	1.3 ± 1	1.8 ± 2	0.3 ± 0.6	1.0 ± 2
	MSI	1.5 ± 1	1.5 ± 2	0.8 ± 2	0 ± 0
	DSI	1.8 ± 0.5	3.3 ± 2	1.4 ± 2	1.6 ± 2
	Col	0.5 ± 0.6	1.3 ± 1	0.4 ± 0.5	1.4 ± 2
Wavy fibers (0–6)	PSI	1.3 ± 1	1.0 ± 1	0.7 ± 1	0 ± 0
	MSI	0 ± 0	0 ± 0	0.8 ± 0.5	0 ± 0
	DSI	1.8 ± 2	2.0 ± 2	0.3 ± 0.5	1.3 ± 1
	Col	3.5 ± 2	4.0 ± 3	1.9 ± 1	2.0 ± 2
Immune infiltration (0–6)	PSI	3.3 ± 2	3.8 ± 2	2.3 ± 1	1.7 ± 0.6
	MSI	2.5 ± 2	4.3 ± 2	2.0 ± 1	1.3 ± 0.6
	DSI	3.5 ± 1	4.5 ± 0.6	2.1 ± 1	2.5 ± 1
	Col	4.0 ± 0	3.7 ± 0.6	1.8 ± 0.7	2.9 ± 2
Vacuolisation (0–6)	PSI	2.5 ± 2	2.0 ± 0.8	1.3 ± 1	2.0 ± 1
	MSI	1.3 ± 2	2.8 ± 1	1.0 ± 0.8	1.7 ± 2
	DSI	1.0 ± 1	1.0 ± 1	2.8 ± 2	2.2 ± 1
	Col	1.0 ± 1	1.0 ± 2	1.0 ± 1	1.8 ± 1

Note

No significant differences are found between PM_2.5_‐exposed animals and their respective controls.

Abbreviations: Col, colon (n_ST_ = 3–4 n_LT_ = 8); DSI, distal small intestine (n_ST_ = 4 n_LT_ = 8); MSI, middle small intestine (n_ST_ = 4 n_LT_ = 3–4); PSI, proximal small intestine (n_ST_ = 4 n_LT_ = 3).

### 3.3 Inflammatory response

**FIGURE 3:**
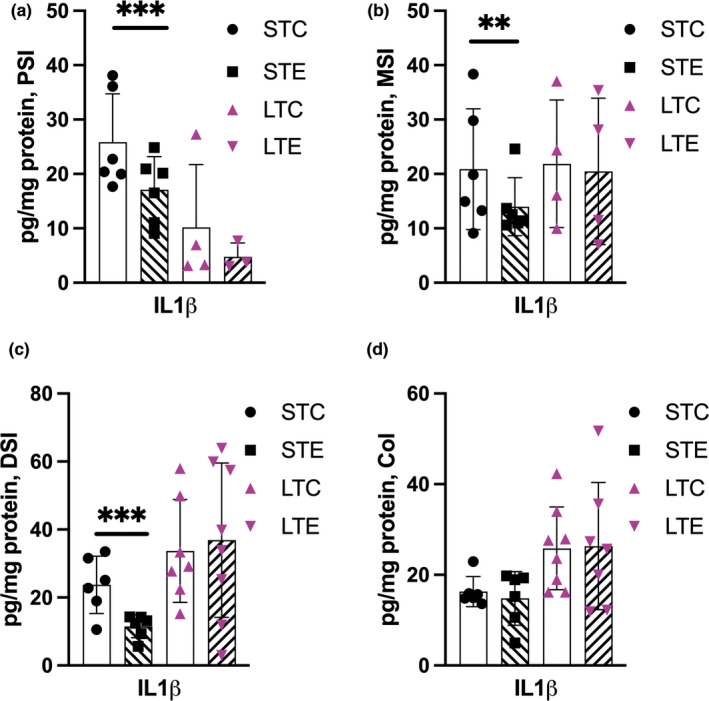
Short‐term PM_2.5_ exposure decreases IL1β in the small intestine. Protein levels of IL1β after short‐term (ST) and long‐term (LT) exposure to PM2.5 dispersed in PBS (STE, LTE indicated with hatched bars) or PBS‐exposed controls (STC, LTCindicated with white bars). (a) Proximal small intestine (PSI), (b) middle small intestine (MSI), (c) distal small intestine (DSI), (d) colon (Col). Data presented as individual values and mean ± SD, ^**^ *p* < 0.01, ^***^ *p* < 0.001

Analyses showed IL1β IL4, IL6, IL10, IL13, TNF, and CXCL1 to be differently expressed along the intestine. With most pronounced differences being observed in IL1β, IL4, IL6, IL10, TNF, and CXCL1 between proximal and distal regions of the intestine in the long‐term control and PM_2.5_‐exposed animals (Figure S2). Further, IL4 (*p* = 0.008), INFγ (*p* = 0.005) were reduced in long‐term control animals compared to short‐term control animals in the PSI (Figure S2b,h). Levels of inflammatory markers in each intestinal segment after short‐ and long‐term PM_2.5_ exposure were compared to their respective controls. IL1β was significantly reduced in all small intestinal areas after short‐term PM_2.5_ exposure (PSI *p* < 0.0001, MSI *p* = 0.006, DSI *p* < 0.0001). IL1β results are presented in Figure 3 and remaining biomarkers in Figure S2.

### 3.4 No changes in enteric neurons after PM_2__.__5_ exposure

**FIGURE 4:**
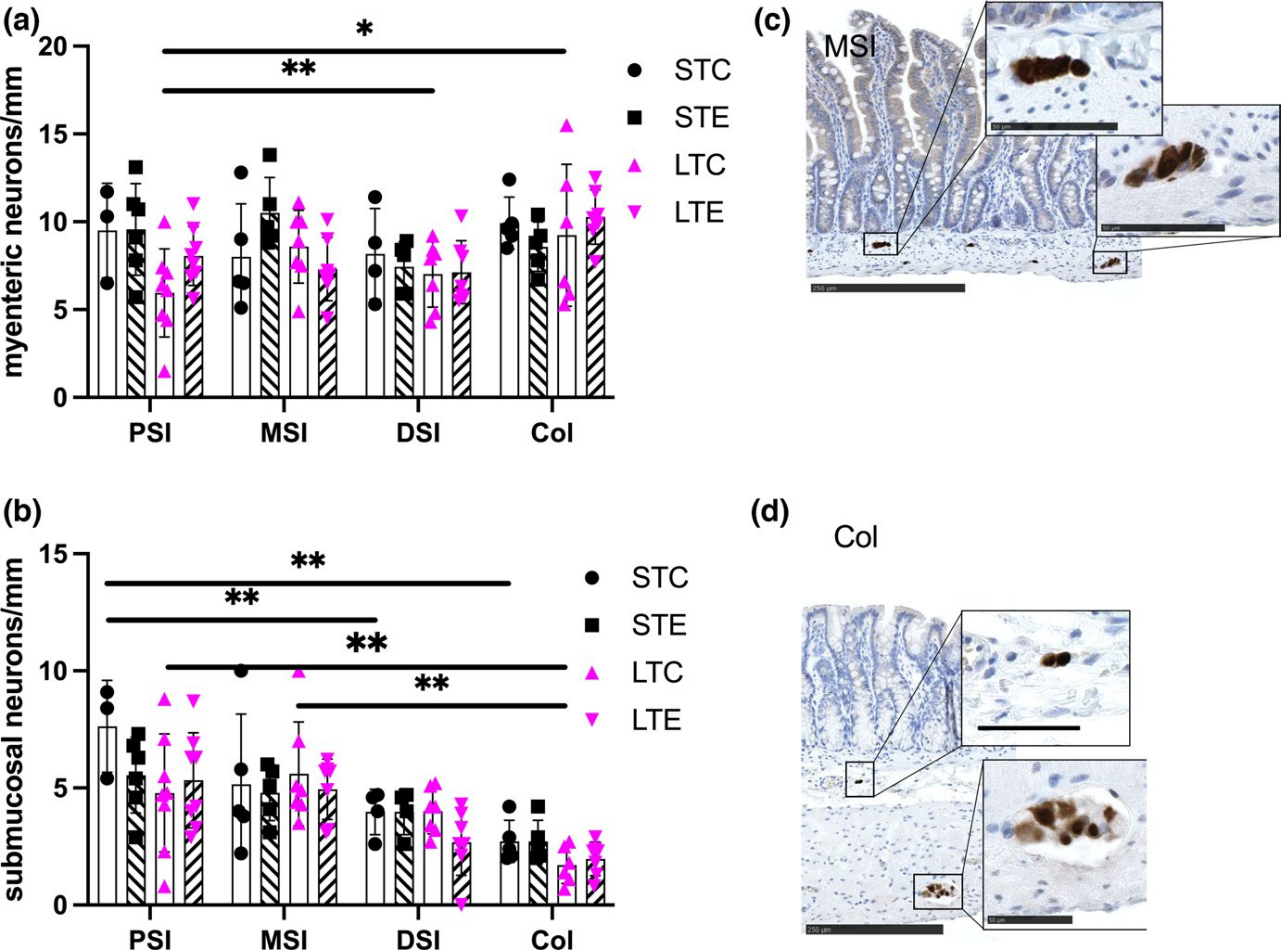
Exposure to PM_2.5_ does not reduce enteric neuronal numbers in the intestine. Density of myenteric (a) and submucosal (b) neurons along the intestine (proximal; PSI, middle; MSI; distal; DSI) and short‐term (ST) and long‐term (LT) exposure to PM_2.5_ dispersed in PBS (STE, LTE indicated with hatched bars) or PBS‐exposed controls (STC, LTC indicated with white bars). Density of enteric neurons changes along the intestine. Representative micrographs of enteric neurons in the middle small intestine (c) and colon (d). Upper inserts depict submucosal neurons and lower inserts myenteric neurons. Data presented as individual values and mean ± SD, ^*^ *p* < 0.05, ^**^ *p* < 0.01. Bar in overview micrographs represents 250 μm, and in inserts 50 μm

Assessment of enteric neurons along the GI tract revealed no effect of PM_2.5_ treatment on neither myenteric nor submucosal neurons. Rather, it was found that enteric neuronal numbers differ in density along the intestinal tract. More myenteric neurons were present in the colon compared to PSI in long‐term control animals (*p* = 0.04). More submucosal neurons were present in the PSI compared to DSI (*p* = 0.03) and Col (*p* = 0.001), in the short‐term control animals. The same pattern was present in long‐term control animals with PSI and MSI showing more submucosal neurons compared to Col (*p* = 0.007, *p* = 0.0006 respectively). Results are presented in Figure 4.

## 4 DISCUSSION

Human studies have shown that inhaled PM_2.5_ triggers significant levels of macrophage‐mediated pulmonary clearance (Möller et al., 2004). Together with ingested air, liquids, foods, mucociliary and hepatobiliary clearance the intestine is exposed to significant amounts of PM (Kreyling, Holzwarth, Haberl, et al., 2017; Vignal et al., 2021). The contribution of intestinal exposure to air pollution has, to date, not been fully explored. The exposure protocol applied in this pilot study did not allow us to measure the specific concentration of delivered dose to each animal. However, the protocol enabled delivery of PM_2.5_ in a physiologically relevant manner, with intestinal exposure achieved through pulmonary circulatory clearance, mucociliary clearance, ingested mist, and grooming. Based on estimations of respiratory volume and mucociliary clearance from the nasal cavity and tracheobronchial space in rats the intestinal load were estimated to approach 82 μg/h or ~646 μg/session in short‐term experiments and 27 μg/h or ~81 μg/session in long‐term experiments. Given the moist conditions grooming may have provided additional PM_2.5_ exposure. Estimation of this volume can be done using the volume of droplets per groomed area. Nebulizers generally produce droplets of 5 μm in diameter or less (Phipps & Gonda, 1990) leading to a droplet volume of ~0.5 nm^3^ and area of 20 μm^2^. A rat weighing 300 g have an estimated total body surface area of 440 cm^2^ (Gouma et al., 2012) leading to a droplet volume of ~115 μl or 0.3 μg–0.8 μg PM_2.5_ accumulated on each animal, numbers relatively marginal compared to the loads estimated to be associated mucociliary clearance.

The PM_2.5_ used in this study contained both a range of metals and PAH’s of which several are known to have the potential to cause metabolic disruption (Nääv et al., 2020). However, our results indicate that sustained long‐term PM_2.5_ exposure did not lead to a significant inflammatory response in the intestine compared to controls. Our data suggest that long‐term exposure leads to an adaptation phenomenon as the short‐term exposure protocol led to higher mucosal lesion scores compared to the control. This adaptation might relate to the comparably low‐dose PM_2.5_ exposure delivered over a longer period. An interesting alternative could be the presence of potential PM_2.5_‐diet interaction. Recently it was shown that people consuming a diet high in whole‐grain, vegetables, fruits, and dairy were less sensitive to pollution associated cardiovascular changes (Xu et al., 2021). The chow diet consumed by animals in this study align with recommendations for laboratory rodent growth and maintenance (Nutrition NRCUSoLA, 1995). This type of diet may have an intestinoprotective index capable of absorbing the pollution associated load present in the long‐ but not short‐term exposure. The observed mucosal lesions were associated with epithelial separation from the basal lamina and the lamina propria mucosae. Epithelial cells are anchored on the basal lamina with anchoring complexes consisting of laminin‐1 and ‐5 together with their respective integrin receptors alpha7beta1 and alpha3beta1 (Beaulieu, 1999; Leivo et al., 1996). Bulk detachment of epithelial cell lining from the basal lamina has been associated with a lack of cytoplasmic integrin support in response to mechanical activation (Ussar et al., 2008). Interestingly, PM_2.5_ exposure has been shown to downregulate genes associated with integrin signaling in human airway epithelial cells (Lan et al., 2021). The intestinal lining is renewed weekly through a process of controlled extrusion known as anoikis (Patankar & Becker, 2020). This process involves loss of contact with the basal lamina by β1 integrins, and has been shown to induce a transient expression of IL1β after detachment (Stadnyk & Kearsey, 1996). Both endo‐ and exogenous produced IL1β are shown to have an anti‐anoikis effect on intestinal epithelial cells in vitro, stimulating pathways promoting cellular aggregation (Waterhouse et al., 2001). Our observations of short‐term PM_2.5_ exposure‐induced epithelial detachment and reduced IL1β. It is, thus, intriguing to speculate that luminal or absorbed PM_2.5_ can affect cell adhesion. It is plausible that both the disruption of anoikis and a reduced IL1β response could lead to an intestinal lining which is less resilient to mechanical stress associated with digestion. This would require further investigation in the future.

Particulate matter has been shown to alter the colonic microbiome in mice (Bailey et al., 2020; Ran et al., 2021) as well as increase IL6 expression, barrier permeability and cellular stress after intragastric delivery of PM_2.5_ (Mutlu et al., 2011). We observed an area specific pattern of inflammatory markers, with the proximal small intestine showing lower levels compared to more distal parts and the colon. Reflecting the different roles and varying microbial loads present along the intestine (Gourbeyre et al., 2015). The inflammatory markers measured in this study were, except for IL1β in the small intestine, unaffected by PM_2.5_ treatment.

Particulates from, for example, diesel exhaust have been shown to induce neuronal damage in vitro (Ji et al., 2019). Moreover, epidemiological evidence indicates that pollution exposure is a risk factor for the development of neurodegenerative diseases (Tsai et al., 2019). A possible entry route into the central nervous system has been suggested to be the olfactory epithelium that shows histopathological changes in response to PM. It is suggested PM is retrogradely transported into the brain and causes neuronal damage (Ajmani et al., 2016). In an accompanying study these animals were found to have increased IL6 expression in cerebral tissue after long‐term exposure (Voss et al., 2022), this was speculated to potentially be secondary to altered vascular function. However, another alternative is retrograde transport from the periphery. Intramuscular injections of bismuth (Stoltenberg et al., 2001), ingested TiO_2_ nanoparticles (Kreyling, Holzwarth, Schleh, et al., 2017) and even small proteins like α‐synuclein (Holmqvist et al., 2014) have been found to be transported to the central nervous system via retrograde axonal transport. The gut is innervated by the enteric nervous system consisting of millions of neurons regulating and controlling intestinal motility, blood flow, and secretion (Furness, 2012). This system is innervated by the autonomic nervous system ensuring bidirectional communication between peripheral and central systems (Furness, 2012). Neuroplastic changes to, or loss of enteric neurons has been associated with inflammatory bowel disorders (Vasina et al., 2006), irritable bowel syndrome (De Giorgio et al., 2004), Parkinson's disease (Leclair‐Visonneau et al., 2020), and stroke (Cheng et al., 2016), several of which have been associated with air pollution (Kasdagli et al., 2019; Liu et al., 2017; Vignal et al., 2021). The interaction between PM_2.5_, enteric neurons, and disease should be further investigated in the future.

In the same accompanying study (Voss et al., 2022), bronchoalveolar lavage from these animals showed a transient increase of leukocytes after short‐ but not long‐term exposure. No effects on serum inflammatory makers were observed either, indicating that it is not one type of inflammation, inflammatory cell, or cytokine response that drives the systemic effects. But rather that extended PM_2.5_ exposure slightly shifts inflammatory reactivity patterns. Further, this lack of significant inflammatory response, which has elsewhere been reported (Falcon‐Rodriguez et al., 2016), was in this study suggested to be explained by the larger droplet size and temperature drop associated with the use of the jet nebulizer. This, in theory, limited lung exposure and need for alveolar macrophages to remove pulmonary particles led to lower systemic exposure and thus systemic inflammation to PM_2.5_. Saturating the atmosphere with mist droplets may increase intestinal exposure compared to dry delivery due to greater nasal and tracheobronchial clearance. Highlighting the need for further exploration of how climate conditions and air pollution interact to impact human health. In this study, an effect between short‐ and long‐term control animals was observed across several of the measured parameters. Whether these effects are associated with differences in age between animals, for example, 2 versus 4 months or potentially due to aerosol exposure is a question that needs to be further explored. PM composition is an amalgamation of both local and distal environmental factors (Nääv et al., 2020), using nebulizers to aerosolize PM into the environment may have affected PM_2.5_ composition in both exposed and control conditions. This may be particularly important since it has been shown that humidifiers increase the concentration of PM in direct correlation to the concentration of solutes and ions present in the water used (Lau et al., 2021). Further highlighting the need for investigations into how variability in PM_2.5_ composition, climate, and delivery methods can contribute to the physiological post‐exposure effects. Effects which can be felt by millions of people globally.

## 5 LIMITATIONS

Several limitations exist in this study, which in future studies should be addressed. This includes better control of the exact load of PM_2.5_ particles delivered to the GIT as well as evaluating effects across different ages and in both sexes. Expanding group sizes to achieve higher power as well as including other control groups to understand the effects of humidity and temperature would have strengthen findings, including evaluating different cellular aspects of the observed histopathology.

## CONFLICT OF INTEREST

The authors declare no conflict of interest.

## 6 Ethical statement

All procedures and animal experiments were performed in full compliance with ARRIVE and the European Community Council Directive (2010/63/EU) for Protection of Vertebrate Animals Used for Experimental and other Scientific Purposes guidelines. Ethical permit was approved by the Malmö‐Lund Institutional Ethics Committee under the Swedish National Department of Agriculture (Animal Inspectorate License No. Dnr M16‐15).

## AUTHOR CONTRIBUTIONS

Conceptualization LO, LU, CI, UV; Methodology LO, CI, UV, LU, LF, SW, WEO, SA; Analyses LF, SW, WEO, UV; Writing original draft LF, SW, WEO, UV, SA, LU, CI, LO; Writing review and editing; SA, UV Supervision UV; Funding acquisition LO, LU, UV.

## Supporting information



Fig S1‐S2
