# Supplementary material for: Short‐term exposure to urban PM2.5 particles induces histopathological and inflammatory changes in the rat small intestine
Source: Physiol Rep. 2022 Apr 13;10(7):e15249. doi: 10.14814/phy2.15249 (PMC9006536; doi:10.14814/phy2.15249)
Supplement: Supplementary file 1 — Fig S1‐S2 [file PHY2-10-e15249-s001.docx]

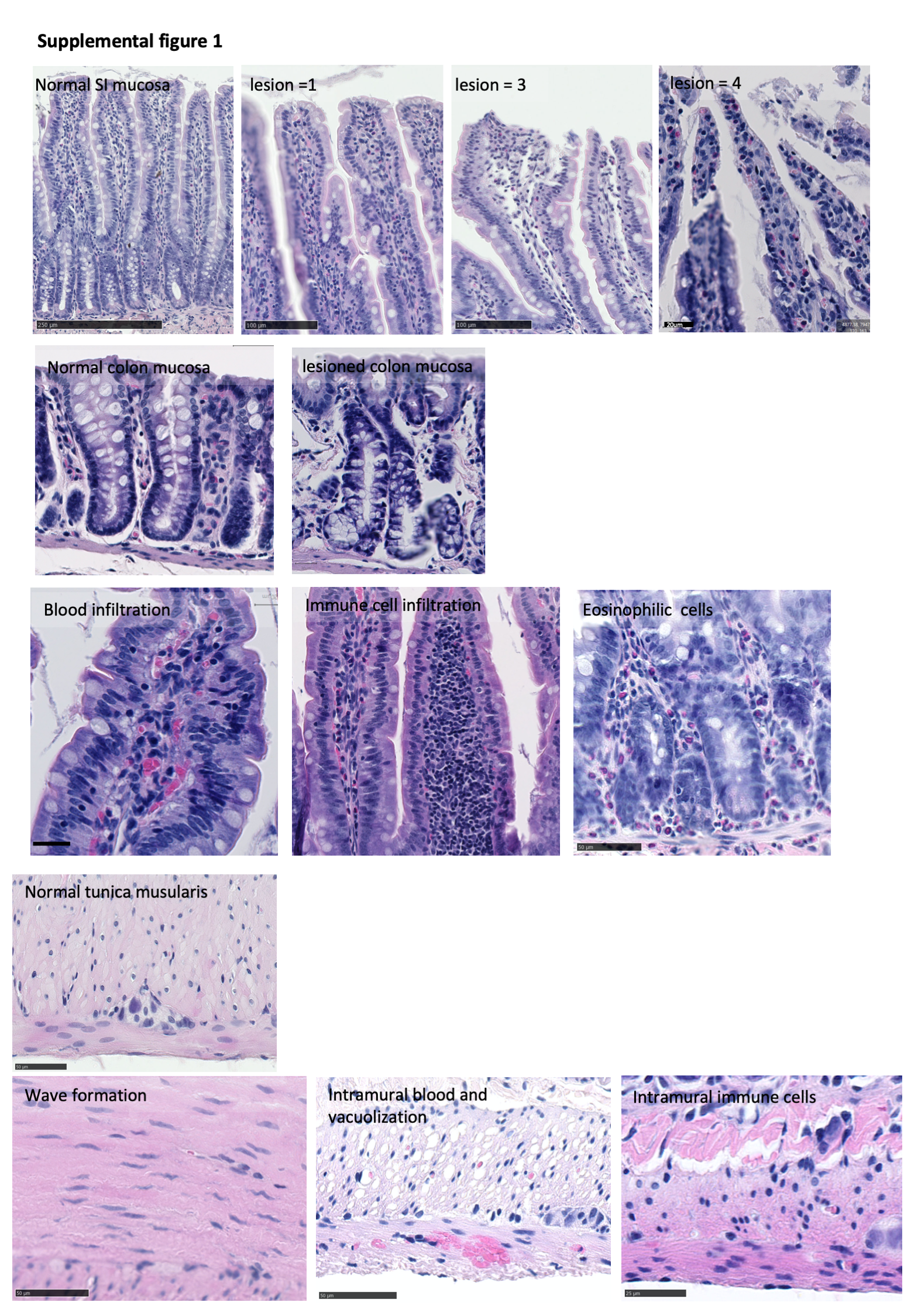


Examples of histopathological lesions, in small intestinal mucosa, colon mucosa and in tunica muscularis.

**Supplemental figure 2**

Levels of measured inflammatory markers along the intestine from proximal small intestine (psi), through middle (msi), distal (dsi) to the colon (col). Long-term control animals show an increasing proximal to distal gradient of IL1β, IL4, IL6, IL10 and TNF levels compared to short-term control animals. No PM2.5 induced changes in measured markers IL4, IL5, IL6, IL10, TNF, CXCL1 and INF-γ are found in neither short-term or long-term PM.25 exposed animals when compared to their respective controls. Data individual values and mean±SD.
